# Validity of Diagnosis Code–Based Claims to Identify Pulmonary NTM Disease in Bronchiectasis Patients

**DOI:** 10.3201/eid2703.203124

**Published:** 2021-03

**Authors:** Jennifer H. Ku, Emily M. Henkle, Kathleen F. Carlson, Miguel Marino, Kevin L. Winthrop

**Affiliations:** Oregon Health & Science University–Portland State University School of Public Health, Portland, Oregon, USA (J.H. Ku, E.M. Henkle, K.F. Carlson, M. Marino, K.L. Winthrop);; Veterans Affairs Portland Healthcare System, Portland (K.F. Carlson)

**Keywords:** bronchiectasis, nontuberculous mycobacterial infection, validation, Medicare claims, bacteria, respiratory infections, tuberculosis and other mycobacteria

## Abstract

Nontuberculous mycobacteria infection is increasing in incidence and can lead to chronic, debilitating pulmonary disease. We investigated the accuracy of diagnosis code–based nontuberculous mycobacteria lung disease claims among Medicare beneficiaries in the United States. We observed that these claims have moderate validity, but given their low sensitivity, incidence might be underestimated.

Nontuberculous mycobacteria (NTM) infection is an illness of increasing incidence caused by environmental organisms and can lead to chronic pulmonary disease (*1**–**5*). The accuracy of International Classification of Diseases (ICD) diagnosis codes for NTM infection has been evaluated only in limited fashion (*6*) and is unknown in the context of bronchiectasis, which most patients with pulmonary NTM infection have (*7**,**8*). We investigated the accuracy of ICD diagnosis codes for NTM infection among Medicare beneficiaries in the United States by using the Bronchiectasis and NTM Research Registry (BRR) as the reference standard.

We identified persons with a diagnosis of bronchiectasis (ICD Ninth Revision, Clinical Modification [ICD-9-CM], codes 494.0 or 494.1) from 2006–2014 Medicare data. BRR is a database of persons with bronchiectasis, NTM infection, or both at 13 US medical institutions (*8*). BRR captures clinical data from the 24-month period before enrollment and at annual follow-ups. We matched study participants enrolled at 7 BRR sites to Medicare data (*9*). Medicare observation began on the later date of either enrollment or data-start (January 1, 2006) and ended on the earlier date of either coverage-end or data-end (December 31, 2014). We included study participants with an overlap in BRR and Medicare observation, excluding claims or cultures outside this overlap.

We established a primary case definition of an NTM infection as >1 inpatient discharge or outpatient visit coded 031.0 (pulmonary mycobacterial infection) assigned by a clinician; we also established alternative definitions (Table). For the primary and each alternative case definition, we calculated positive predictive value (PPV) as the proportion of Medicare claim–based NTM infections meeting the BRR case definition +12 months of the first claim. Sensitivity was calculated as the proportion of patients meeting the BRR case definition who had a claim for NTM infection within +12 months of meeting that definition. All analyses were performed by using SAS statistical software 9.4 (SAS Institute Inc., https://www.sas.com). This study was approved by the Institutional Review Board at Oregon Health & Science University.

**Table:** Positive predictive value and sensitivity of ICD-9-CM diagnosis code–based case definitions for NTM infection in 2006–2014 Medicare data by using Bronchiectasis and NTM Research Registry as reference standard, United States*

NTM case definition†	No. participants with diagnosis-based Medicare claim for NTM infection	PPV (95% CI)‡	No. participants meeting BRR case definition for NTM infection§	Sensitivity (95% CI)¶
Primary definition: ICD-9-CM 031.0				
All clinician-given codes#	234	63.2 (57.1–69.4)	226	69.9 (63.9–75.9)
ID specialist– and pulmonologist-given codes only	205	65.4 (58.9–71.9)	226	61.5 (55.2–67.9)
ID specialist–given codes only	127	70.1 (62.1–78.0)	226	39.8 (33.4–46.2)
Pulmonologist-given codes only	133	60.9 (52.6–69.2)	226	36.7 (30.4–43.0)
Secondary definition: ICD-9-CM 031.0, requiring a second 031.0 claim >30 d but <12 m of first claim				
All clinician-given codes	122	72.1 (63.3–79.9)	226	41.6 (35.2–48.0)
ID specialist– and pulmonologist-given codes only	100	74.0 (64.3–82.3)	226	33.2 (27.1–39.7)
ID specialist–given codes only	45	82.2 (71.1–93.4)	226	16.4 (11.6–21.2)
Pulmonologist-given codes only	44	70.5 (57.0–83.9)	226	13.3 (30.4–43.0)

*BRR, Bronchiectasis and NTM Research Registry; NTM, nontuberculous mycobacteria; ICD-9-CM, International Classification of Disease, Ninth Version, Clinical Modification; ID, infectious disease; PPV, positive predictive value.  †Only ICD-9-CM 031.0 code (pulmonary mycobacterial infection) was considered; other codes for NTM (031.8 [other specified mycobacterial diseases] and 031.9 [unspecified disease due to mycobacteria]) were not considered. ‡PPV for meeting a case definition for NTM infection in BRR within +12 months of first ICD-9-CM NTM code-cased claim (code 031.0). §NTM cases were identified in BRR on the basis of culture positivity on >1 respiratory specimen or antibiotic treatment for NTM during follow-up (a macrolide plus >1 antibiotic drugs). ¶Sensitivity for an ICD-9-CM NTM code–based claim within +12 months of meeting a case definition for NTM infection in BRR. #Clinician types include physicians, physician assistants, and nurse practitioners, excluding radiology or laboratory-associated claims.

Of the 530 Medicare beneficiaries also enrolled in BRR at the 7 sites, 457 (86.2%) were matched (Figure). Our final analytic sample included 403 participants who averaged 73.5 years of age (range 62–98 years, SD 6.2) and were mostly women (80.4%) and white (95.8%). Of the 403 participants, 205 (50.9%) had >1 NTM infection claim based on a diagnosis code assigned by a clinician. 

**Figure:**
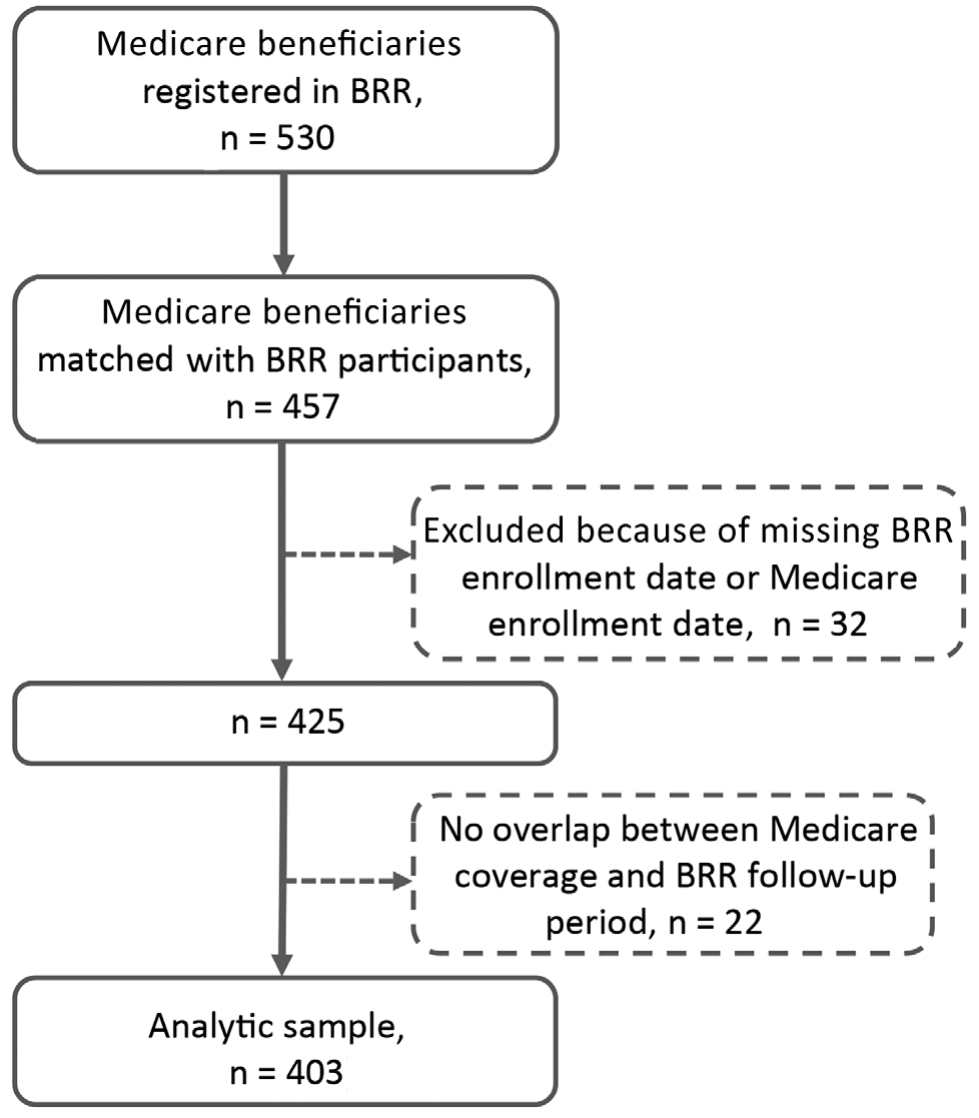
Flow diagram of the analytic sample (n = 403) of Medicare beneficiaries and persons from BRR matched during 2006–2014, United States. Original pool of Medicare beneficiaries (n = 530) included beneficiaries of Medicare parts A, B, and D but not C and excluded those with cystic fibrosis and a history of HIV or organ transplant. BRR, Bronchiectasis and NTM Research Registry.

We observed that diagnosis code–based claims have moderate validity for identifying NTM infection. Our primary case definition had a PPV of 63.2% (95% CI 57.1%–69.4%) (Table) and was 69.9% (95% CI 63.9%–75.9%) sensitive in detecting NTM infection within ±12 months of the first claim date. PPV was maximized when a second claim was required and codes restricted to those assigned by an infectious disease specialist. In a previous study, the microbiologic NTM infection case definition (*1*) had a high PPV (77%) and yielded maximized sensitivity and PPV when combined with ICD-9-CM codes (*6*). Our results were similar in that NTM infection codes had fairly high PPVs but lower sensitivity.

False-positive diagnosis codes could be caused by several factors. The Medicare population includes persons with chronic illness whose records might include codes from previous NTM infections, but we could not evaluate this possibility because of limited claims data before BRR baseline. More than half of study participants with false-positive codes had negative cultures, indicating that the code was applied for NTM evaluation or monitoring in the absence of active disease. Higher PPVs, when restricted to specialist-assigned codes, imply that general clinicians might be more likely to assign the disease code when disease criteria are not met. The poor sensitivity was not unexpected; NTM infection is frequently underdiagnosed and miscoded as a nonpulmonary NTM or other infection. Our case definition required 1 positive culture, whereas current diagnostic guidelines require 2; of study participants meeting our case definition, 35% had a second positive culture within 12 months.

A limitation of our study is that we only included Medicare beneficiaries >65 years of age with bronchiectasis; also, BRR collects data from specialized NTM centers, which might differ from general clinic settings. Our Medicare data ended in 2014, limiting the sample size and overlap with BRR observation time. Last, we only evaluated ICD-9-CM codes, although ICD Tenth Revision, Clinical Modification (ICD-10-CM), codes have been required since 2015 (*10*). However, understanding the validity of ICD-9-CM codes is essential for interpretation of the existing literature that is based on ICD-9-CM codes and to inform future research using ICD-10-CM codes. Further, ICD-9-CM codes for NTM map directly to ICD-10-CM codes (ICD-9-CM 031.0 equates to ICD-10-CM A31.0 [pulmonary mycobacterial infection]), helping guide future comparisons.

Our results indicate that a case definition of >2 claims given 30 days apart within 12 months of each other accurately identifies pulmonary NTM infection in patients who also have bronchiectasis. Given low sensitivity, incidence might be severely underestimated in claims-based epidemiologic research. Claims data provide critical information about the epidemiology of NTM infection when clinical data are not available, but findings should be interpreted with awareness of the potential for misclassification.
